# Retinitis pigmentosa with optic neuropathy and *COQ2* mutations: A case report

**DOI:** 10.1016/j.ajoc.2022.101298

**Published:** 2022-01-20

**Authors:** Kentaro Kurata, Katsuhiro Hosono, Masakazu Takayama, Masahisa Katsuno, Hirotomo Saitsu, Tsutomu Ogata, Yoshihiro Hotta

**Affiliations:** a Department of Ophthalmology, Hamamatsu University School of Medicine, 1-20-1 Handayama, Higashi-ku, Hamamatsu, 431-3192, Japan; b Department of Neurology, Nagoya University Graduate School of Medicine, 65 Tsurumai, Syowa-ku, Nagoya, 466-8560, Japan; c Department of Biochemistry, Hamamatsu University School of Medicine, 1-20-1 Handayama, Higashi-ku, Hamamatsu, 431-3192, Japan; d Department of Pediatrics, Hamamatsu University School of Medicine, 1-20-1 Handayama, Higashi-ku, Hamamatsu, 431-3192, Japan

**Keywords:** Retinitis pigmentosa, Optic neuropathy, *COQ2* gene, Targeted next-generation sequencing

## Abstract

**Purpose:**

To report the clinical findings of a Japanese patient presenting with retinitis pigmentosa (RP) together with optic neuropathy and *COQ2* mutations.

**Observations:**

The patient had experienced night blindness and photophobia since his 20s. At 27 years of age, he experienced sudden vision loss in his left eye. We performed comprehensive ophthalmic examinations. Trio-based whole-exome sequencing was performed to identify the candidate variants, which were confirmed by Sanger sequencing. Fundus examination revealed typical RP findings with an additional Leber hereditary optic neuropathy (LHON). The patient's visual acuity was severely affected, and the visual field showed central scotoma. Electroretinograms were non-recordable under scotopic condition and showed reduced response under photopic conditions. Genetic analysis revealed compound heterozygous *COQ2* variants in the patient: c.469C > T [p.(P157S], and c.518G > A [p.(R173H)]. Co-segregation analysis in the unaffected parents confirmed that the two variants were in trans. During the 4-year follow-up period, his visual acuity and central scotoma gradually improved.

**Conclusion:**

This is the first description of a case of RP together with LHON harboring *COQ2* mutations. Additional cases are necessary to more accurately determine the clinical course and mutation spectrum in this condition.

## Highlights

F0D7 A novel case of RP with LHON-like optic neuropathy is presented.
F0D7 Genetic analysis suggests that *COQ2* mutations caused RP and optic neuropathy.
F0D7 This case suggests that LHON is caused not only by mtDNA mutations.

## 1 Introduction

Retinitis pigmentosa (RP; OMIM #268000) is an inherited retinal disease that affects 1 in 3000–5000 individuals worldwide.^1^ It presents with progressive visual dysfunction, including night blindness, visual field constriction, and eventual central visual loss. Funduscopic findings include bone spicule retinal pigmentations, chorioretinal atrophy, attenuated retinal vessels, and waxy pallor optic disc. Electroretinograms (ERG) often show a severely reduced or nondetectable response. To date, over 80 disease-causing genes have been associated with RP.

Leber hereditary optic neuropathy (LHON; OMIM #535000) is a maternally inherited disease resulting in optic nerve atrophy^2^ It occurs primarily in young men and is usually caused by mutations in mitochondrial DNA (mtDNA). It usually presents as subacute loss of central vision in one eye, followed by fellow eye involvement weeks to months later. On fundus examination, hyperemic optic disc with peripapillary telangiectasias was commonly seen.

Here, we report a case involving a patient with RP presenting with an additional LHON and showing compound heterozygous mutations in *COQ2* (coenzyme Q2, polyprenyltransferase) on whole-exome sequencing (WES).

## 2 Case report

All procedures were approved by the Institutional Review Board for Human Genetic and Genome Research at the Hamamatsu University School of Medicine (permit no. 14–040). All study procedures adhered to the guidelines of the Helsinki Declaration. Written informed consent was obtained from the patient and parents after detailed information of the procedures were explained. Before and after undergoing genetic examination, the patient received genetic counseling.

### 2.1 Clinical assessment

The patient, a 27-year-old Japanese man with non-consanguineous parents, was examined at the Hamamatsu University School of Medicine. He underwent comprehensive ophthalmic examinations, including best-corrected visual acuity (BCVA) measurement, refraction assessment, slit-lamp biomicroscopy, dilated ophthalmoscopy, kinetic visual field assessment with Goldmann perimetry (GP), fundus photography, and fluorescein angiography. Central retinal laminar architecture was evaluated by optic coherence tomography (OCT; Cirrus HD-OCT; Carl Zeiss Meditec, Dublin, CA). Full-field ERGs were obtained in accordance with the protocols of the International Society for Clinical Electrophysiology of Vision.^3^

### 2.2 Genetic analyses

Genomic DNA was extracted using the QIAamp DNA Blood Midi Kit (Qiagen, Hilden, Germany) according to the manufacturer's instructions. Trio samples (one affected individual and unaffected parents) were analyzed by WES. Exome data processing, variant calling, and variant annotation were performed as previously described,^4^^,^^5^ by using Human GRCh38 as the reference genome. To identify the disease-causing variants, we focused on nonsynonymous variants and splice-site variants, which are within 10 bp of the exon-intron boundaries (±10 bp), and excluded synonymous and non-coding exonic variants from the analysis. We treated common genetic variants (allele frequency >0.01 for recessive variants or >0.001 for dominant variants) in any of the ethnic subgroups found in the following single nucleotide polymorphism databases and in-house exome data (n = 218) as putative non-pathogenic sequence alterations: Genome Aggregation Database (gnomAD; https://gnomad.broadinstitute.org/), Human Genetic Variation Database (HGVD; http://www.genome.med.kyoto-u.ac.jp/SnpDB/), and Integrative Japanese Genome Variation Database (4.7KJPN; https://ijgvd.megabank.tohoku.ac.jp/). Particular attention was paid to variants in known causative genes associated with inherited retinal dystrophy (http://www.sph.uth.tmc.edu/Retnet/. Accessed on June 23, 2020).

Potential pathogenic variants detected by WES were validated using Sanger sequencing according to the standard protocol.^6^ Sanger sequencing segregation analyses were performed in the three family members to investigate the co-segregation of potentially pathogenic variants. The following primer set for the *COQ2* was used: exon 2 of *COQ2*: forward primer 5ʹ -AGTAAGGGGTCCTTTGTGATTTG-3ʹ, and reverse primer 5ʹ- CTGTGGTCACTGAATGATCTTGTT-3ʹ. NCBI Reference Sequences of *COQ2* (NM_015697.8) was used.

### 2.3 Clinical findings

**Fig. 1:**
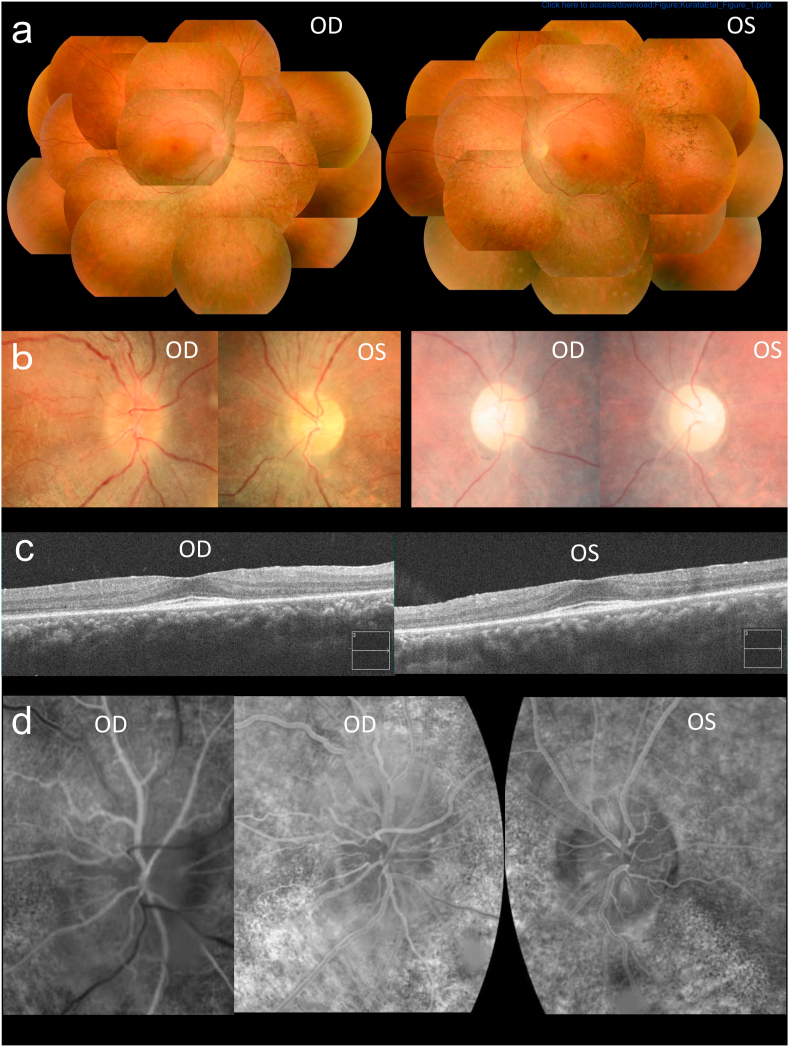
Fundus findings of the patient (a) Color fundus photography at the first visit showed diffuse retinal degeneration, including bone-spicule pigmentation and narrowed retinal vessels. (b) The optic disc was hyperemic in the right eye and pale in the left eye at the first visit (left row). The optic discs were pale in both eyes at the last visit (right row). (c) Optical coherence tomography at the first visit revealed relatively preserved inner retinal layers. Thinning of the outer nuclear layer and disruption of the ellipsoid zone were seen from the parafoveal area to the surrounding area. (d) Fluorescein angiography at the first visit revealed peripapillary telangiectatic blood vessels in the early phase in the right eye (left row) and neither staining nor leakage around the optic disc in the late phase in both eyes (middle and right row). OD, oculus dexter; OS, oculus sinister. .(For interpretation of the references to color in this figure legend, the reader is referred to the Web version of this article.)

**Fig. 2:**
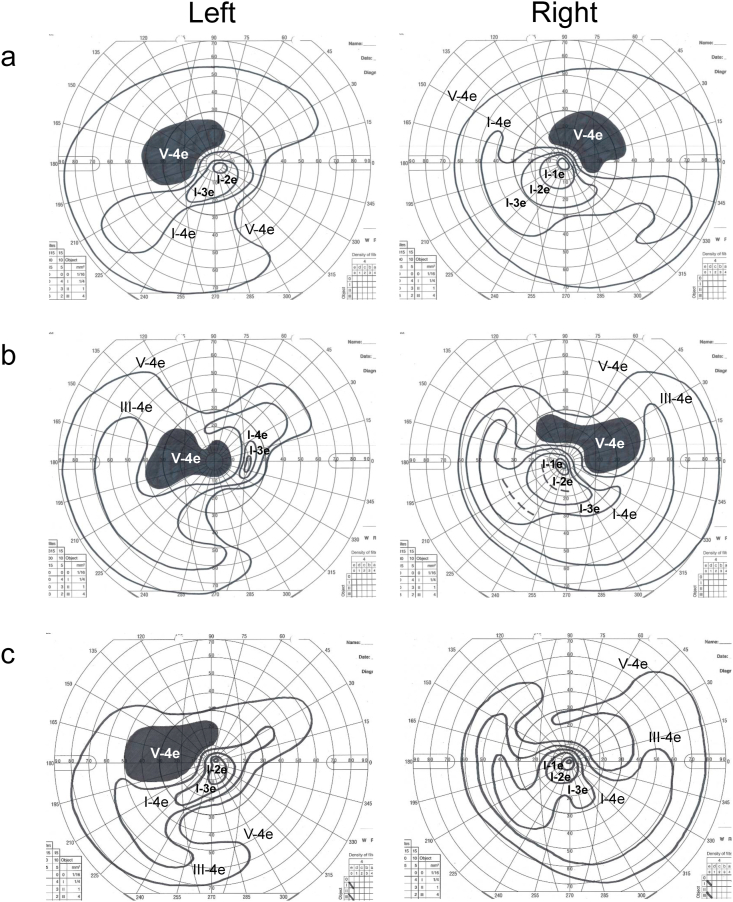
Goldmann perimetry (GP) findings of the patient before and after sudden visual acuity loss (a) GP examination before sudden visual acuity loss showed arcuate scotoma in both eyes. (b) GP examination after sudden visual acuity loss showed enlarged arcuate scotoma in both eyes and central scotoma in his left eye. (c) Central scotoma in his left eye had disappeared.

**Fig. 3:**
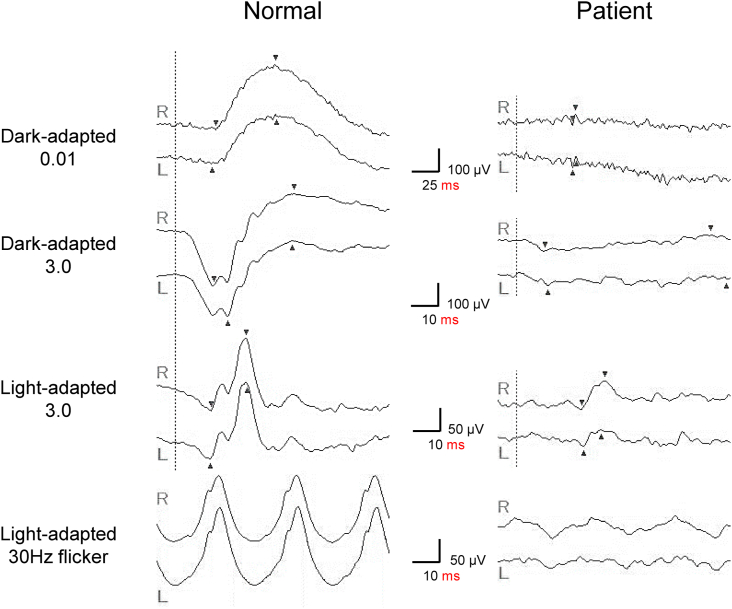
Electroretinograms (ERGs) of the patient ERGs of both eyes were non-recordable under scotopic conditions and showed reduced response under photopic conditions.

**Fig. 4:**
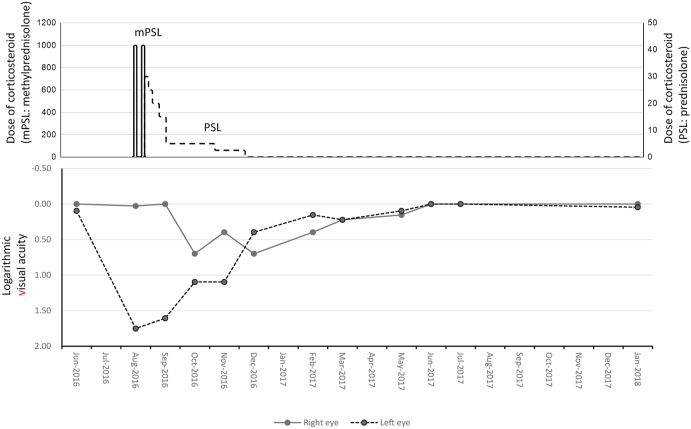
Course of best-corrected visual acuity in the patient The graph shows visual acuity values expressed in logMAR units. Visual loss in the right and left eyes occurred subacutely with a gap of time, eventually improving spontaneously.

The patient had been experiencing night blindness and photophobia since his 20s and was diagnosed as having RP. His family members, including parents, grandparents, and siblings, were not affected. He experienced sudden visual loss in his left eye a month ago and received an ophthalmic examination at a town doctor's clinic. Optic disc pallor, central scotoma, and abnormal color vision in his left eye, and hyperemic optic disc in his right eye were seen. Although optic neuropathy was suspected and he received steroid pulse therapy, an obvious effect could not be obtained. He was referred to our hospital. He also reported recent visual loss in his right eye. His BCVA converted to logarithm of the minimum angle of resolution (logMAR) was 0 in the right eye and 1.7 in the left eye. The anterior segments were normal. Fundus examination revealed diffuse retinal degeneration together with bone-spicule pigmentation and narrowed retinal vessels (Fig. 1a). The optic disc was hyperemic in his right eye and pale in his left eye (Fig. 1b). The visual field on GP showed paracentral scotoma in his right eye and central scotoma in his left eye (Fig. 2a and b). ERGs were non-recordable under scotopic conditions and showed reduced response under photopic conditions (Fig. 3). OCT demonstrated relatively preserved inner retinal layers (Fig. 1c). The foveal pit seemed to be absent because apparent thinning of the outer nuclear layer and disruption of the ellipsoid zone were seen except at the foveal region. Fluorescein angiography revealed no staining or leakage around the optic disc in the late phase in both eyes, and peripapillary telangiectatic blood vessels were detected in his right eye, consistent with LHON (Fig. 1d). He had no relevant medical history, used no medicines, and was not exposed to toxins or illicit drugs. Blood examination showed that autoantibodies, including aquaporin-4-antibody, were absent, and vitamin B12 levels were within the normal limit. Magnetic resonance imaging revealed no intracranial abnormalities. After consulting with the patient, additional steroid pulse therapy was performed. However, his visual function did not improve immediately after steroid pulse therapy. Two months later, his right logMAR BCVA decreased to 0.7. However, over a four-year period, BCVA and the visual field gradually improved, and the central scotoma in his left eye disappeared (Fig. 4). At the current examination, his logMAR BCVA was 0 in the both eyes. Optic discs pallor, paracentral scotoma, and color disturbance in both eyes were seen (Fig. 1, Fig. 2c). On physical examination, neither renal dysfunction nor neurological abnormalities were detected. Thrombocytopenia was found (platelet count, 30 × 10^9^/L; reference values 153–352 × 10^9^/L) on blood examination. He was negative for anti-platelet antibody and a bone-marrow aspirate examination did not reveal any abnormalities. Therefore, the thrombocytopenia was diagnosed as idiopathic thrombocythemia.

### 2.4 Genetic studies

To elucidate the pathogenesis, we performed WES using trio samples. We performed analyses based on the autosomal recessive and *de novo* dominant models. No rare variants were detected in known causative genes associated with inherited retinal dystrophy (http://www.sph.uth.tmc.edu/Retnet/. Accessed on June 23, 2020) or mtDNA associated with *LHON.* Consequently, we identified two heterozygous *COQ2* variants in the patient: c.469C > T [p.(P157S)], and c.518G > A [p.(R173H)]. Co-segregation analysis in the unaffected parents confirmed that the two variants were in trans. The c.469C > T and c.518G > A variants in *COQ2* have previously been reported as a cause of multiple system atrophy.^7^^,^^8^ According to the guideline of the American College of Medical Genetics and Genomics,^9^ both variants were classified as likely pathogenic (Table). While other rare *de novo*, hemizygous, and compound heterozygous variants were identified, there were no data to support of the relevance of such variants to our patient's phenotypes (Supplementary table).

## 3 Discussion

The patient reported night blindness and visual field defect due to RP and presented with a sudden central visual acuity defect, which was considered to be due to optic neuropathy. Optic neuropathy in the right and left eyes occurred subacutely with a time gap, and was steroid-resistant, eventually improving spontaneously (Fig. 4). Although the optic disc was hyperemic at the initial examination, it later became pale. Fluorescein angiography revealed peripapillary telangiectatic blood vessels without staining or leakage around the optic disc. These findings resemble that of LHON.^10^ Although the visual prognosis in LHON is generally poor, there are some reports of improvement of visual function.^11^

*COQ2* is considered to encode the biosynthetic pathway of coenzyme Q10 (CoQ10), and *COQ2* mutations can cause CoQ10 deficiency.^12^ In addition to *COQ2*, seven genes are reported to be associated with CoQ10 deficiency in humans: *PDSS1*, *PDSS2*, *COQ4*, *COQ6*, *ADCK3*, *ADCK4*, and *COQ9*.^13^, ^14^, ^15^, ^16^, ^17^, ^18^, ^19^ CoQ10 acts as an electron shuttle in the mitochondrial respiratory chain and associates to synthesize adenosine triphosphate (ATP).^20^ Therefore, lack of CoQ10 can cause failure of electron transfer, resulting in respiratory chain dysfunction and decreased ATP synthesis. In addition, because CoQ10 functions as an antioxidant and removes various reactive oxygen species in cells, decreased CoQ10 levels can cause vulnerability to oxidative stress.^21^ Deficiency of CoQ10 manifests with various clinical presentations. For example, in the case of *COQ2* mutation, multiple system atrophy, encephalomyopathy, ataxia, lactic acidosis, deafness, RP, optic atrophy, myopathy, and steroid-resistant nephrotic syndrome have been reported with various combinations.^7^^,^^12^^,^^22^, ^23^, ^24^, ^25^ Two siblings (71 and 66 years old) presented with multiple system atrophy and RP^7^ A 33-month-old boy suffering from optic atrophy in addition to encephalomyopathy and nephropathy has been reported^12^ However, the detailed ocular features were not described in these reports. To our knowledge, there have been no reports of *COQ2* mutations associated with ocular abnormalities only without systemic abnormalities. In our case, although systemic abnormalities, such as renal or neurological disorders, were not observed, careful observation is needed in the future. Rötig et al. reported a patient with CoQ10 deficiency demonstrating RP and optic atrophy, but the causal gene was not examined.^26^ The mechanism through which CoQ10 deficiency causes RP is uncertain. Mansergh et al. reported a large Irish kindred of RP caused by mtDNA mutation.^27^ Considering the above reasons, mitochondrial dysfunction due to either CoQ10 deficiency or mtDNA mutation can exhibit an RP phenotype.

LHON generally occurs due to mutations in the genes of the mitochondria, which induce apoptosis of retinal ganglion cells by decreasing ATP synthesis and elevating oxidative stress.^28^ Although the typical manifestation of LHON is only optic neuropathy, other manifestations such as cardiac, neurologic, and skeletal abnormalities have been reported.^29^ Only one previous report has described LHON complicated with RP, in which mutations in *RP2* and mtDNA were detected.^30^ In our case, rare variants were not detected in the known causative genes associated with inherited retinal dystrophy or mtDNA associated with LHON. Given that the database for known genes associated with inherited retinal dystrophies is certainly not 100%, the possibility that this patient may also have harbored a separate set of mutations in a gene that has not yet been associated with RP or LHON cannot be ruled out. Therefore, it is speculated that *COQ2* mutation cause CoQ10 deficiency, resulting in mitochondrial electron transfer system dysfunction and decreased ATP synthesis, followed by the occurrence of LHON in addition to RP.

The limitation of this study is that neither the proportion of mitochondrial respiratory chain complexes nor the CoQ10 level was measured. Moreover, functional analysis was not performed. If the CoQ10 level in tissue or cells was found to be low, CoQ10 supplementation may have improved the visual disturbance in this patient. Fortunately, in our case, spontaneous visual improvement was obtained.

In conclusion, this is the first report of an RP case together with optic neuropathy harboring *COQ2* mutations. Although it cannot be ruled out that this patient may also harbor a separate set of mutations in a gene that has not yet been associated with RP or LHON, it is speculated that *COQ2* mutations cause mitochondrial electron transfer system dysfunction, leading to RP and LHON. Long-term follow-up is required to define the ultimate prognosis of this patient. Additional cases are necessary to more accurately determine the clinical course and mutation spectrum in this condition.

## Patient consent

Written informed consent was obtained from the patient and parents for publication of this case report and any accompanying images.

## Funding

This work was supported by a grant for the Initiative on Rare and Undiagnosed Diseases (no. JP18ek0109301) from the 10.13039/100009619Japan Agency for Medical Research and Development and the 10.13039/501100001691Japan Society for the Promotion of Science Grant-in-Aid for Scientific Research (C) (nos. JP17K11447 to Y.H. and JP16K11284 to K.H.).

## Authorship

All authors attest that they meet the current ICMJE criteria for Authorship.

## Declaration of competing interest

The authors declare that there are no conflicts of interest regarding this paper.

## References

[1] Chizzolini M., Galan A., Milan E., Sebastiani A., Costagliola C., Parmeggiani F. (2011). Good epidemiologic practice in retinitis pigmentosa: from phenotyping to biobanking. Curr Genom.

[2] Wallace D., Singh G., Lott M. (1988). Mitochondrial DNA mutation associated with Leber's hereditary optic neuropathy. Science.

[3] McCulloch D.L., Marmor M.F., Brigell M.G. (2015). ISCEV Standard for full-field clinical electroretinography (2015 update). Doc Ophthalmol.

[4] Hayashi T., Hosono K., Kubo A. (2020). Long-term observation of a Japanese mucolipidosis IV patient with a novel homozygous p.F313del variant of MCOLN1. Am J Med Genet A.

[5] Hiraide T., Nakashima M., Yamoto K. (2018). De novo variants in SETD1B are associated with intellectual disability, epilepsy and autism. Hum Genet.

[6] Hosono K., Ishigami C., Takahashi M. (2012). Two novel mutations in the EYS gene are possible major causes of autosomal recessive retinitis pigmentosa in the Japanese population. PLoS One.

[7] Multiple-System Atrophy Research Collaboration (2013). Mutations in COQ2 in familial and sporadic multiple-system atrophy. N Engl J MedN Engl J Med.

[8] Chen Y.P., Zhao B., Cao B. (2015). Mutation scanning of the COQ2 gene in ethnic Chinese patients with multiple-system atrophy. Neurobiol Aging.

[9] Richards S., Aziz N., Bale S. (2015). Standards and guidelines for the interpretation of sequence variants: a joint consensus recommendation of the American College of medical genetics and Genomics and the association for molecular pathology. Genet Med.

[10] Newman N.J., Lott M.T., Wallace D.C. (1991). The clinical characteristics of pedigrees of Leber's hereditary optic neuropathy with the 11778 mutation. Am J Ophthalmol.

[11] Hotta Y., Hayakawa M., Fujiki K. (1993). An atypical Leber's hereditary optic neuropathy with the 11778 mutation. Br J Ophthalmol.

[12] Quinzii C., Naini A., Salviati L. (2006). A mutation in *para*-hydroxybenzoate-polyprenyl transferase (COQ2) causes primary coenzyme Q10 deficiency. Am J Hum Genet.

[13] Mollet J., Giurgea I., Schlemmer D. (2007). Prenyldiphosphate synthase, subunit 1 (PDSS1) and OH-benzoate polyprenyltransferase (COQ2) mutations in ubiquinone deficiency and oxidative phosphorylation disorders. J Clin Invest.

[14] López L.C., Schuelke M., Quinzii C.M. (2006). Leigh syndrome with nephropathy and CoQ10 deficiency due to decaprenyl diphosphate synthase subunit 2 (PDSS2) mutations. Am J Hum Genet.

[15] Salviati L., Trevisson E., Rodriguez Hernandez M.A. (2012). Haploinsufficiency of COQ4 causes coenzyme Q10 deficiency. J Med Genet.

[16] Heeringa S.F., Chernin G., Chaki M. (2011). COQ6 mutations in human patients produce nephrotic syndrome with sensorineural deafness. J Clin Invest.

[17] Horvath R., Czermin B., Gulati S. (2012). Adult-onset cerebellar ataxia due to mutations in CABC1/ADCK3. J Neurol Neurosurg Psychiatry.

[18] Ashraf S., Gee H.Y., Woerner S. (2013). ADCK4 mutations promote steroid-resistant nephrotic syndrome through CoQ10 biosynthesis disruption. J Clin Invest.

[19] Duncan A.J., Bitner-Glindzicz M., Meunier B. (2009). A nonsense mutation in COQ9 causes autosomal-recessive neonatal-onset primary coenzyme Q10 deficiency: a potentially treatable form of mitochondrial disease. Am J Hum Genet.

[20] Turunen M., Olsson J., Dallner G. (2004). Metabolism and function of coenzyme Q. Biochim Biophys Acta.

[21] Kagan V., Serbinova E., Packer L. (1990 June 29). Antioxidant effects of ubiquinones in microsomes and mitochondria are mediated by tocopherol recycling. Biochem Biophys Res Commun.

[22] Diomedi-Camassei F., Di Giandomenico S., Santorelli F.M. (2007). COQ2 nephropathy: a newly described inherited mitochondriopathy with primary renal involvement. J Am Soc Nephrol.

[23] Jakobs B.S., van den Heuvel L.P., Smeets R.J. (2013). A novel mutation in COQ2 leading to fatal infantile multisystem disease. J Neurol Sci.

[24] Doimo M., Desbats M.A., Cerqua C., Cassina M., Trevisson E., Salviati L. (2014). Genetics of coenzyme q10 deficiency. Mol Syndromol.

[25] Quinzii C.M., Hirano M. (2010). Coenzyme Q and mitochondrial disease. Dev Disabil Res Rev.

[26] Rötig A., Appelkvist E.L., Geromel V. (2000). Quinone-responsive multiple respiratory-chain dysfunction due to widespread coenzyme Q10 deficiency. Lancet.

[27] Mansergh F.C., Millington-Ward S., Kennan A. (1999). Retinitis pigmentosa and progressive sensorineural hearing loss caused by a C12258A mutation in the mitochondrial MTTS2 gene. Am J Hum Genet.

[28] Sadun A.A., La Morgia C.L., Carelli V. (2011). Leber's hereditary optic neuropathy. Curr Treat Options Neurol.

[29] Orssaud C. (2018). Cardiac disorders in patients with Leber hereditary optic neuropathy. J Neuro Ophthalmol.

[30] Mashima Y., Saga M., Hiida Y., Imamura Y., Kudoh J., Shimizu N. (2000). Novel mutation in RP2 gene in two brothers with X-linked retinitis pigmentosa and mtDNA mutation of Leber hereditary optic neuropathy who showed marked differences in clinical severity. Am J Ophthalmol.

